# Complement Inhibition Therapy and Dialytic Strategies in Paroxysmal Nocturnal Hemoglobinuria: The Nephrologist’s Opinion

**DOI:** 10.3390/jcm9051261

**Published:** 2020-04-26

**Authors:** Guido Gembillo, Rossella Siligato, Valeria Cernaro, Domenico Santoro

**Affiliations:** Unit of Nephrology, Department of Clinical and Experimental Medicine, University of Messina, 98125 Messina, Italy; rossellasiligato@gmail.com (R.S.); valecern82@virgilio.it (V.C.); dsantoro@unime.it (D.S.)

**Keywords:** paroxysmal nocturnal hemoglobinuria, complement inhibition, eculizumab, ravulizumab, hemodialysis

## Abstract

Paroxysmal nocturnal hemoglobinuria (PNH) is a rare clonal disease that presents an estimated incidence of 1.3 cases per million per year, with a prevalence of 15.9 cases per million. It is characterized by hemolysis, bone marrow dysfunction with peripheral blood cytopenia, hypercoagulability, thrombosis, renal impairment and arterial and pulmonary hypertension. Hemolysis and subsequent hemosiderin accumulation in tubular epithelium cells induce tubular atrophy and interstitial fibrosis. The origin of PNH is the somatic mutation in the X-linked phosphatidylinositol glycan class A (PIG-A) gene located on Xp22: this condition leads to the production of clonal blood cells with a deficiency in those surface proteins that protect against the lytic action of the activated complement system. Despite the increased knowledge of this syndrome, therapies for PNH were still only experimental and symptomatic, until the introduction of the C5 complement blockade agent Eculizumab. A second generation of anti-complement agents is currently under investigation, representing future promising therapeutic strategies for patients affected by PNH. In the case of chronic hemolysis and renal iron deposition, a multidisciplinary approach should be considered to avoid or treat acute tubular injury or acute kidney injury (AKI). New promising perspectives derive from complement inhibitors and iron chelators, as well as more invasive treatments such as immunoadsorption or the use of dedicated hemodialysis filters in the presence of AKI.

## 1. Introduction

Paroxysmal nocturnal hemoglobinuria (PNH) is a rare clonal disease that presents an estimated incidence of 1.3 cases per million per year, with a prevalence of 15.9 cases per million [1].

The name of this disease is due to the characteristic severe hemolytic anemia giving episodes of hemoglobin in the urine, particularly evident in overnight urine concentrations, but that happen all day long.

The main features of PNH are represented by hemolysis, bone marrow dysfunction, hypercoagulability, thrombosis and smooth muscle dystonia, all signs and symptoms that can also cause particularly severe complications such as renal failure and arterial and pulmonary hypertension [2,3]. If not treated, PNH could lead to death in approximately 35% of affected people within five years of diagnosis and 50% within 10 years of diagnosis [4]. A major complication of PNH, when complement inhibition therapy is delayed or not available, is Acute Kidney Injury (AKI), even though different authors have reported in literature their clinical experience in courses of severe renal impairment and there is not a clear and univocal strategy universally accepted.

Continuous Renal Replacement Therapy (CRRT) is one of the best choices for the treatment of PNH-associated AKI, but an appropriate choice of dialyzers and convective dose could be the real key to determining the most appropriate treatment. The contribution of the nephrologist should be to make an accurate choice of dialytic prescription, taking into account the variety of filters and dialysis modalities available.

If the CRRT is an important tool in the eventuality of acute renal impairment, the use of complement inhibitors could also guarantee a maintenance therapy in order to reduce the possibility of disease flares.

Eculizumab [5] has represented, during the past few years, the main agent directed against this disease, but recently, a growing number of complement inhibitors has been under investigation, such as Ravulizumab [6], new generations of C5 inhibitor, Coversin [7], Compstatin analogs and C3 Inhibitors [8] or Factor D inhibitors [9]. This second generation of anti-complement agents represents future promising therapeutic strategies for patients affected by PNH.

## 2. PNH Etiopathology, Laboratory Findings and Clinical Manifestations

### 2.1. PNH Genetic Mutation

The cause of PNH is a somatic mutation in the X-linked phosphatidylinositol glycan class A (PIG-A) gene on Xp22 [10], coding for one of the several enzymes involved in the generation of glycosyl phosphatidylinositol (GPI) anchors in the endoplasmic reticulum. Deficiency in *PIG A* leads to a lack of the expression of approximately 150 cell surface proteins [11]. The common core structure of GPI is made up of a molecule of phosphatidylinositol and a glycan core consisting of three mannoses, an ethanolamine phosphate and glucosamine [12].

At least 10 reactions and more than 20 different genes are implicated in the biosynthesis of GPI anchors [13]. In PNH, this mutation results in the production of clonal blood cells with a deficiency in those surface proteins that protect against damage caused by the complement system [14]. In this way, such a disorder makes these cells excessively susceptible to the lytic action of activated complement.

**Figure 1:**
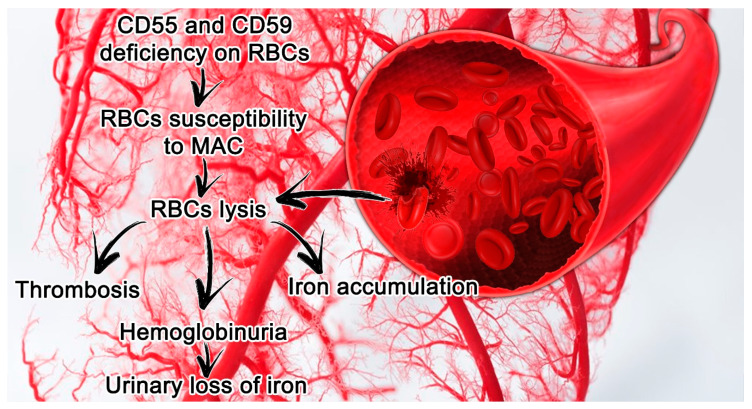
Paroxysmal nocturnal hemoglobinuria (PNH) features in small vessels and its consequences. Erythrocytes lacking CD55 and CD59 are more susceptible to hemolysis mediated by MAC, which leads in turn to thrombosis and the release of hemoglobin and free iron. Abbreviations: CD55, a decay accelerating factor; CD59, a membrane inhibitor of reactive lysis; RBC, red blood cells; MAC, membrane attack complex.

PNH features are closely connected with a deficit in or complete absence of CD55 (a decay accelerating factor) and CD59 (a membrane inhibitor of reactive lysis), from the family of GPI-anchored proteins. These proteins modulate solid phase complement activity; CD55 inhibits alternative pathway C3 and C5 convertases, and CD59 prevents the creation of the membrane attack complex (MAC) [15] (Figure 1).

Other proteins have a minor role in PNH pathophysiology, the most common of which are monocyte differentiation antigen (CD14), low-affinity immunoglobulin gamma Fc region receptor III-B (CD16b) and CD48 antigen [16,17,18].

### 2.2. PNH Laboratory Findings and Clinical Manifestations

The major clinical manifestations and complications of PNH are associated with hemolytic anemia, thrombosis and bone marrow failure [19].

Laboratory results in PNH patients highlight anemia with negative Coombs tests and hemoglobinuria with dipsticks positive for heme but negative sediments for red blood cells, increased reticulocyte counts, elevated levels of lactate dehydrogenase (LDH) and bilirubin, diminished levels of haptoglobin, and iron deficiency. Diagnosis is based on the demonstration of the PNH phenotype in a substantial proportion of red cells and granulocytes [20].

PNH patients show various degrees of anemia and other bone marrow-related disorders such as granulocytopenia and thrombocytopenia. The incidence of anemia ranges from 88% to 94% and that of leukopenia ranges from 41% to 72%, while that of thrombocytopenia ranges from 51% to 80% [21,22].

Anemia derives from both hemolysis and bone marrow dysfunction that is mostly intravascular [23]. Even if hemolysis is continuous during the day, in the night-time, the decline in the blood pH triggers an activation of complement components. This mechanism may be also dependent on an increased nocturnal absorption of lipopolysaccharide (LPS), a major component of the outer membrane of bacteria that stimulates the complement system and is normally bound by monocytes through a GPI-linked protein, CD14, which is lacking in PNH patients [24,25,26]. The urine is black when the patient awakens, because the amount of free hemoglobin passing through the glomeruli exceeds the absorptive ability of renal tubules [27].

Thrombosis represents one of the major determinants of morbidity and mortality in PNH patients [28]. In particular, thromboembolism is the principal cause of mortality in this population, leading to 40%–67% of deaths for which the cause is known [29]. PNH venous thrombosis often occurs at uncommon sites such as hepatic veins, causing Budd–Chiari syndrome, which represents the principal (40.7%) thrombotic event in these subjects. The second most frequent type of thrombosis is represented by cerebral vein and sinus thrombosis. Pulmonary embolism represents a high-risk site of thrombosis and has been reported in 26 cases, 10 of which were fatal [30].

Arterial thrombosis has been described in a minority of subjects, mostly of young age, supporting the hypothesis that it may occur de novo without significant predisposing atherosclerotic disease [31].

The pathophysiology of the pro-thrombotic state in PNH is still debated. Ploug et al. [32] supposed a failure of the fibrinolytic system, such as a deficiency in urokinase type plasminogen activator receptor on leucocytes. A significant role can be attributed to dysregulated complement activity on GPI anchor-deficient platelets, granulocytes, monocytes and endothelial cells [33]. Complement-mediated attack on CD55 and CD59 deficient platelets stimulates the production of factors Va, Xa and prothrombin complex [34].

The most-adopted PNH classification was proposed in 2005 by the International PNH Interest Group [35], which identified three different kinds of clinical manifestation: classic PNH, which includes patients who have evidence of PNH without bone marrow disorder; PNH associated with another bone marrow disorder [36]; and subclinical PNH, in which patients have defective PNH clones without clinical or laboratory signs of hemolysis or thrombosis. De Latour et al. [37] added a fourth subgroup defined as intermediate PNH, underlining the fact that many PNH patients do not perfectly fit into these categories because they may suffer from cytopenia or signs of a still-undiagnosed underlying bone marrow failure.

## 3. PNH and Kidney Diseases

### 3.1. PNH and Chronic Kidney Failure

According to Hillmen et al. [38], who analyzed a cohort of 195 PNH patients, 65% of their population suffered from chronic kidney disease (CKD) and approximately 21% had a moderate-to-severe renal impairment (CKD Stage 3 to 5).

Kim et al. [39] similarly reported that more than half PNH patients could develop kidney disease and so that renal function should be systematically monitored after PNH diagnosis. The incidence of renal events reported in their study was comparable with the 45% observed in the Spanish PNH Registry [40]. Interestingly, Nishimura et al. [22] assessed that renal failure also contributed to 8%–18% of total PNH-related deaths.

### 3.2. Renal Hemosiderosis in the Course of PNH

Zachee et al. [41] described a case of a PNH patient with CKD and hemosiderosis who had received multiple blood transfusions for severe anemia, thus raising the possibility that transfused iron load could contribute to renal hemosiderosis.

The exact process of iron accumulation associated with kidney injury has not been entirely explained yet. Ferrous iron catalyzes the Fenton process, thanks to its peculiarities as an electron donor: through this catalytic reaction, hydrogen peroxide is converted into a highly cytotoxic hydroxyl free radical. Experimental hemosiderosis animal models demonstrated that increased lipid peroxidation is linked with iron overload and subsequent deposition in the kidney, with an increment in both plasma and renal tissue malondialdehyde, a lipid peroxidation product, and an increment in the expression of renal endothelial and inducible nitric oxide synthase [42,43]. Rubio-Navarro et al. [44] showed that podocytes bind and endocytose Hb in vitro and in vivo, resulting in increased oxidative stress and morphological changes in podocytes such as cell hypertrophy, actin cytoskeleton rearrangement and apoptosis [45]. Hemoglobin (Hb) also decreases the expression of nephrin and synaptopodin, two multifunctional proteins that maintain the structural integrity of the slit diaphragm and glomerular filtration function [46], partially explaining the PNH urinary manifestation in the course of Hb oxidative injury.

Hb renal uptake activates nuclear factor erythroid-2-related factor 2 (Nrf2); this mechanism was identified in mice with intravascular hemolysis and podocyte injury and loss. Moreover, Hb induces the production of the Nrf2-related antioxidant proteins heme oxygenase 1 (HO-1) and ferritin.

HO enzymes have also a central role in the degradation of heme and its inducible isozyme, HO-1, which has a protective role versus acute heme protein-induced kidney toxicity [47]. HO-1’s functional importance in AKI is shown by multiple studies in which its action was experimentally altered, leading to a worsened response to ischemia/reperfusion damage [48].

However, HO’s contribution is still controversial: despite positive effects being observed in several cells and organs [49], various studies support the hypothesis that increased HO-1 expression may contribute to the promotion of other pathogenic conditions such as cancer [50].

### 3.3. PNH and Renal Tubulopathies

Hemolysis and subsequent hemoglobinuria lead to a progressive hemosiderin deposition in the proximal convoluted tubules. There, hemosiderin cannot be filtrated due to its high molecular weight and accumulates in the epithelium, causing tubular atrophy and interstitial fibrosis [43]. Tubular injury may also induce Fanconi’s syndrome, characterized by an altered renal reabsorption of small molecules, resulting in hyper-aminoaciduria, glycosuria, hyperphosphaturia and bicarbonaturia [51,52] and in the concurrent dysfunction of distal H^+^ secretion. Moumas et al. [53] reported two cases of Fanconi’s syndrome with diffuse hemosiderin inclusions within proximal tubular cells. Magnetic resonance imaging showed an abnormal kidney iron load, which was significantly reduced after treatment with Eculizumab.

Hsiao et al. [54] described a case of renal hemosiderosis due to chronic hemolytic episodes and iron overload in a patient with PNH treated with multiple transfusions. Fanconi’s syndrome could be identified and should also be suspected in patients with other chronic hemolytic disorders and chronic illnesses requiring repeated blood transfusions.

Hemoglobin (Hb) dimers released from red blood cells upon hemolysis are internalized by megalin/cubilin receptors on tubular cells, which are particular sensitive to heme toxicity [55]. Free heme triggers the transcription factor Nuclear Factor kappa B (NF-κB) in the epithelial cells of the proximal tubule. NF-κB activation causes the metabolic reprogramming of and phenotypic changes in immune and parenchymal cells, as well as the secretion of several inflammatory mediators in the interstitium, which exacerbate interstitial nephritis and the irreversible loss of function [56,57].

Acute kidney injury (AKI) secondary to endogenous compounds such as heme may be diagnosed from a wide spectrum of lesions at renal biopsy, ranging from loss of the brush border, cytoplasmic vacuolation and cellular swelling to the extensive necrosis of tubular cells. Regenerative changes such as flattening of the lining, cytoplasmic basophilia, a higher nuclear-to-cytoplasmic ratio in individual cells, and cellular mitoses can also be detected [58]. Electron microscopy underlines the loss of proximal tubular brush borders and shows diminished infoldings at the basolateral membrane of proximal tubular epithelial cells (PTECs) [59]. At the same time, renal hemosiderosis is a common finding, with irregularly shaped, dark granular deposits grouped in a ring-like structure with a pale center in tubules, as well as chronic tubulointerstitial injury [60,61].

Renal hemosiderosis is not only a rare cause of AKI; it can also induce chronic kidney failure [62]. Asim et al. [63] reported a case of detailed evidence of CKD progressing over a few years, associated with pancytopenia, in a patient who had no previous admissions for hemolytic crisis or AKI. His only relevant findings at renal biopsy were hemosiderosis, interstitial fibrosis and tubular atrophy, and he was later diagnosed with PNH.

Kümpers et al. [52] documented a case of a PNH patient who developed AKI on CKD after a hemolytic crisis subsequent to an episode of intercurrent urinary tract infection. The patient presented diffused renal siderosis in magnetic resonance imaging, confirmed at kidney biopsy, which also revealed acute tubular necrosis and global glomerular sclerosis.

### 3.4. Glomerular Damage in the Course of PNH

Glomeruli can also be involved in PNH as shown by many case reports. Gupta et al. [64] presented a case of CD59 deficiency with PNH-like features in a patient with rapidly progressive renal failure due to C1q Nephropathy. Boqari et al. [65] also reported a case of PNH associated with C1q nephropathy, hypothesizing the possible pathogenesis of glomerular C1q deposition in the setting of PNH. A reduced expression of CD59 may be associated with decreased C1q–calreticulin interaction. Calreticulin is a multifunctional protein located in the endoplasmic reticulum and cell membrane of a variety of cells, including red blood cells. This protein has also been identified on apoptotic and necrotic cell surfaces and is considered to play a role in immunogenic cell death and other extracellular functions [66]. It is possible that, in their patient, a lack of CD59 was responsible for both intravascular hemolysis and an excess of unbound C1q in the circulation, resulting in glomerular C1q deposition, opening new suggestive scenarios in the pathogenesis of the disease.

Additionally, IgA nephropathy has been associated with PNH in two case reports [67,68]. Kato et al. described a rare and problematic case of PNH complicated by IgA nephropathy and AKI in the Eculizumab pre-era: in this case, kidney biopsy revealed the focal and segmental proliferation of mesangial cells, mesangial matrix expansion, acute tubular necrosis, interstitial fibrosis and hemosiderin deposits within the tubular epithelial cells, with immunofluorescence microscopy positive for IgA, C3 deposition in the mesangium and an electron microscopy that revealed electron dense deposits in the mesangial area, presenting heavy electron-dense hemosiderin pigments in PTECs [68]. Kuto et al. [69] and Cozzi et al. [70] described two cases of adult Henoch–Schönlein purpura with glomerulonephritis and PNH with several hemolytic episodes.

Takahashi et al. [71] presented the only case reported in the literature of PNH combined with focal segmental glomerular sclerosis.

Another rare association is the one reported by Lee et al. [72], who described a PNH patient with concomitant a Membranous Nephropathy (MN) manifestation of chronic graft-versus-host-disease subsequent to a transplantation of non-myeloablative stem cells.

### 3.5. Renal Compliance in the Course of PNH Therapy

At present, iron overload may be controlled by chelation therapy, which exerted a marked decrease in morbidity and mortality among these patients [73].

The most used iron chelators that could be used in this condition are deferoxamine (DFO), deferiprone and deferasirox, which, unfortunately, present unfavorable pharmacokinetics and pharmacodynamics and nonspecific tissue distribution and can cause significant adverse effects [74]. New opportunities emerged with the use of nanochelators that, after a single-dose intravenous injection in rats, showed a kidney-specific biodistribution and rapid renal excretion (>80% injected dose in 4 h) compared to native (DFO), also significantly reducing the kidney damage caused by iron overload, without demonstrating DFO’s own nephrotoxicity [75].

The most significant development in PNH was represented by the approval of eculizumab, a humanized monoclonal antibody that inhibits terminal complement activation, targeting hemolysis [76]. This agent radically modified the symptoms, biology and natural history of PNH, ameliorating the life conditions of people with PNH [77,78].

Kanakura et al. [79] evaluated the efficacy and safety of eculizumab in a cohort of 29 Japanese PNH patients, of which 66% had CKD. Eculizumab treatment ameliorated CKD in 41% of the patients at 12 weeks, thus suggesting that treatment with eculizumab may have long-term beneficial effects on renal function in PNH patients.

## 4. Acute Kidney Injury

### 4.1. Acute Kidney Injury and Dialysis Choices in PNH—A Nephrologist’s Opinion

The link between PNH and the kidney has been increasingly investigated and validated through its modern history [80,81]. One of the main features of PNH is intravascular hemolysis, which can be chronic, resulting in iron deposition in the kidneys [82], often accompanied by severe hemolytic episodes causing massive hemoglobinuria and AKI, probably from (ATN) development [83] (Table 1).

In Kidney Disease Improving Global Outcome (KDIGO) clinical practice guidelines [84], AKI is defined as any of the following: an increase in serum creatinine (sCr) by ≥0.3 mg/dL (≥26.5 µmol/L) within 48 h; an increase in serum creatinine to ≥1.5 times baseline that is known or presumed to have occurred within the preceding 7 days; or a urine volume <0.5 mL/kg/h for 6 h [85].

The definition and staging of acute kidney injury are mainly based on the risk, injury, failure, loss, end-stage kidney disease (RIFLE) criteria [86] and acute kidney injury network (AKIN) criteria [87].

In PNH, AKI can be a consequence of intravascular hemolysis from the increased destruction of red blood cells and release of free hemoglobin [88]. A similar manifestation can be found in all those conditions where hemosiderin deposits induce acute tubular necrosis: in those cases, upon the hemolysis of erythrocytes, hemoglobin alpha-beta dimers are liberated and filtered by glomeruli, causing hemoglobinuria [89].

Hemoglobin dimers are absorbed by renal proximal tubular cells and degraded, and free iron ions that could be chelated are stored as hemosiderin in the lysosomes [90].

Hemoglobin present in the kidneys secondary to hemolysis could drive the formation of casts mixed with Tamm Horsfall protein (THP) [91]. THP is a relevant immunomodulatory molecule that represents a valid biomarker of urinary system diseases [92], also being a molecule efficient in defending against infections and renal damage-induced inflammation [93,94].

Intravascular hemolysis in PNH patients induces nitric oxide reduction and endothelial and smooth muscle dysregulation, with consequent vasculopathies and arterial hypertension. Intense hemolysis also causes the release of arginase into the plasma from erythrocytes: it converts l-arginine into ornithine, thus decreasing the principal fount for Nitric Oxide (NO) production. The lack of NO induces vasoconstriction, especially in the kidney medulla, leading to severe and prolonged hypoxia and tubular necrosis [95]. Hemolysis has been linked to extracellular arginase that reduces the substrate available to NO synthase, endogenous inhibitors of NO synthase activity, and the dysregulation of hemostatic mechanisms [96].

Ballarin et al. [97] hypothesized that the membrane protein CD163, located on the tissue macrophage surface, may have a central role in AKI control during PNH. They reported the case of a patient presenting with an episode of AKI occurring in the course of acute hemolysis. Renal biopsy showed a strong immunostaining of CD163: it is instantly expressed when Hb is released from erythrocytes during physiological or pathological hemolysis, having a scavenger role for the Hb-haptoglobin complex [98]. The uptake of Hb by CD163 not only attenuates the toxic effects of cell-free Hb but also produces anti-inflammatory feedback, such as interleukin-10 release and the synthesis of HO-1 [99], which catalyzes the conversion of heme to biliverdin, decreasing the production of reactive oxygen species (ROS) [100].

Alkaline diuresis was successfully induced in several AKI cases with intravenous sodium bicarbonate (NaHCO_3_), furosemide fluid infusion and the correction of urinary pH, resulting in a full restitution of renal function. Alkaline urine promotes the solubility of pigments, thus limiting cast and crystal formation. At the same time, a higher pH impairs Hb degradation into heme and free iron and their associated nephrotoxic effects. Moreover, increased diuresis, stimulated by both higher hydration and furosemide, reduces pigment deposition in distal convoluted tubules, even if this molecule has the disadvantage of lowering urinary pH [101,102].

A retrospective analysis from the Spanish PNH Registry [40] showed that in patients affected by both AKI and CKD, eculizumab is an essential tool in the treatment of PNH when it is available and the clinical conditions permit its use. Indeed, after eculizumab treatment had been initiated, renal function was ameliorated and preserved with long-term sustained therapy, probably by iron-renal-clearance as well as by the inhibition of the production of anaphylatoxin C5a, the protein fragment released from cleavage of C5. These data, together with previous published studies [103], confirm that early treatment with eculizumab can prevent further episodes of AKI and slow down CKD progression.

When AKI does not respond to pharmacological treatment, renal replacement therapy (RRT) can correct acid–base and electrolyte imbalance, improve volume overload and remove uremic toxins in PNH patients. Different dialytic strategies may be employed: intermittent hemodialysis (IHD) was initially conceived as a treatment for AKI, while CRRT was developed as an alternative when IHD was not tolerated or contraindicated. CRRT can guarantee a more stable hemodynamic condition than IHD, improving survival expectations and preserving the organ function of treated patients [104].

Khajehdehi [105] described a case of PNH associated with severe acute renal failure that required 24 sessions of hemodialysis (HD) over 8 weeks due to prolonged oliguria. The patient was also treated with hemotransfusions, oral iron, folic acid supplementation and prednisolone, fully recovering from the acute event. Nair et al. [59] reported two cases of AKI: the first patient was appropriately hydrated and started prednisone therapy while the second was treated with packed red blood cell transfusion and required two HD sessions, but both had a normal restoration of kidney function.

Balwani et al. [106] presented the case of a female young patient that had two episodes of AKI within a short span of time—with features suggestive of intravascular hemolytic anemia, which eventually led to PNH diagnosis—and was treated with transfusions, oral corticosteroids and dialysis. Kirsch et al. [107] treated AKI with ATN secondary to PNH with HD sessions, with renal function recovery to normal levels. Nishimoto et al. [108] described a case of postpartum AKI in a woman who was diagnosed with PNH through a Ham test and flow cytometry analysis. Renal magnetic resonance imaging showed features typical of renal cortical hemosiderosis. In this case, the patient was successfully treated with HD, plasma exchange and the administration of corticosteroids.

### 4.2. Dialysis Strategies for AKI in PNH Patients

PNH-related AKI is due to the interaction and aggregation of hemolysis products, Tamm–Horsfall protein and the production of tubular casts causing intratubular obstruction and damage, inducing an inflammatory response, interstitial nephritis and fibrosis. The appropriate dialysis strategy for the treatment of this kind of injury could be convective dialysis, even if randomized controlled trials or appropriate investigations or guidelines have been not established in PNH patients.

In order to remove large molecular weight solutes and some protein-bound uremic toxins, high- and medium-cutoff membranes, convective therapy and protein adsorptive membranes are tested choices [109]. Solute removal is strictly linked to the diffusion and convective transport mechanisms associated with a proper treatment prescription and to the features of the filter used during the HD session. The membranes should guarantee the balanced removal of larger toxins with the prevention of protein leakage, more convection and larger exchange volumes and should ensure safety when exposing patients to large quantities of fluids [110].

The choice of the HD filter is a crucial decision to guarantee the best possible outcome. The filter consists of three principal units: the blood compartment, the membrane and the dialysate compartment. The correct balance of their interaction is the key to the final result, and diffusive and convective mechanisms are the principal ways in which to obtain the desired solute removal. The European Union Toxin Working Group classified uremic retentive toxins and solutes into three groups: small solutes with a molecular weight (MW) < 500 Da, protein-bound uremic toxins and middle molecules with a MW ≥ 500 Da [111].

The opportunity of convective technique use overcomes the limitations of the diffusive transport of larger molecular weight solutes by a mechanism that “pulls” them across the membrane by solvent drag transport, thanks to plasma ultrafiltration [112]. Transport by the convection mechanism is also dependent on the specific solute sieving coefficient and on membrane permeability. The sieving coefficient is defined as the ratio between the solute concentration in the filtrate (CF) and the solute concentration in plasma water (CP) in the absence of a diffusion gradient across the membrane: S = CF/CP [113,114]. The standards for sieving coefficient measurement in vitro are represented by two international criteria, ISO 8637 [115] and EN 1283 [116].

Solute removal during a convective RRT is influenced by the processes of secondary membrane formation or membrane fouling, caused by the adhesion of plasma proteins; and of concentration polarization, a mechanism related to the accumulation of rejected compounds at the blood–membrane interface during filtration [117].

The backfiltration mechanism represents a crucial concept to fully understand the role of ultrafiltration in the removal of medium-high solutes. During HD treatment with a high-flux dialyzer, there is a large drop in blood compartment axial pressure that leads to a blood compartment pressure lower than the dialysate compartment pressure. This mechanism, in addition to the oncotic properties of plasma proteins in blood compartment, induces a passage of the ultrafiltrate from the dialysate to the blood, in the opposite direction with respect to the blood–dialysate direction. This backfiltration system is one of the main processes by which larger compounds are removed during standard high-flux HD [118,119].

This concept leads to the process of “internal hemodiafiltration”, a mechanism that happens during HD with high-flux prescription.

The sieving coefficient and diluting effect of the dialysate flow provoke a reduction in the concentration of a particular molecule, removing it in the proximal part of the high-flux dialyzer. In the distal segment of the dialyzer, when a portion of the dialysate is re-infused back into the blood as backfiltrate, the amount of solute re-infused by solvent drag is consistently smaller in comparison with that removed in the proximal part of the dialyzer owing to the blood–dialysate concentration difference, even if the filtration and backfiltration rates are similar.

The use of membranes with major permeability, larger pore size and decreased hollow-fiber inner diameter amplifies the internal filtration during the HD session, improving the clearance of large-solute compounds [120]. In the proximal section of the dialyzer, this mechanism causes an extensive convection effect; at the same time, backfiltration will compensate in the distal part. There is no need for complicated equipment to achieve the desired results, except for a blood flow of at least 300 mL/min with a dialysate flow of 500 mL/min or higher [121].

Several investigations tried to represent the performance of dialyzers in the course of simulated treatments [122,123,124,125,126,127,128]. Boschetti-De-Fierro et al. [129] compared the clearances of middle and large molecules using Theranova (Baxter, Deerfield, IL, USA), Elisio 17H (Nipro, Osaka, Japan), FX CorDiax 80 and FX CorDiax 800 (both Fresenius, Bad Homburg vor der Höhe, Germany.) dialyzers.

Theranova devices demonstrated in vitro clearances that were higher than those reached with Elisio (Nipro, Osaka, Japan) and CorDiax (Fresenius, Bad Homburg vor der Höhe, Germany). Theranova dialyzers clearly reduced the concentration of the highest molecular marker during the test, whereas neither Elisio nor CorDiax induced an appreciable concentration change in the pool during simulated HD. The clearance values for simulated hemodiafiltration (HDF) treatments with Elisio and CorDiax were higher than those given in HD treatments, showing the advantages of highly convective therapies to achieve a better removal of large molecules. However, Theranova dialyzers achieved higher clearance values in HD mode than high-volume HDF for most molecules.

Another future alternative for current dialysis strategies in the course of AKI in PNH is represented by Coupled Plasma Filtration Adsorption (CPFA), a detoxification technique combining a plasma adsorption circuit with a CRRT. The circuit is made of a plasma filter, a resin-adsorbent cartridge and a hemofilter [130]. CPFA consists of a four-pump modular device (Lynda, Bellco^®^, Mirandola, Italy) with a plasma filter (0.45 m^2^ polyethersulfone, cut-off of 800 kDa), a nonselective hydrophobic resin cartridge (140 mL) with a surface of approximately 700 m^2^/g, and a synthetic, high-permeability hemofilter (1.4 m^2^ polyethersulfone) that causes, in postdilution, a convective exchange of the whole blood [131].

As a first step, blood is prediluted with a replacement fluid to prevent clotting in the circuit. Secondly, a fraction of plasma is separated from blood with a plasma filter and then runs through the adsorbing cartridge, designed to non-selectively remove almost all of the pro- and anti-inflammatory mediators and endotoxins. Subsequently, plasma rejoins the rest of the blood passing through a standard hemofilter. Postdilution replacement fluid is eventually added to the purified blood, which is re-infused into the patient [132]. The use of different techniques leads to a more accurate removal of molecules responsible for the pathophysiology of the patient’s disease, restoring electrolyte and acid–base equilibrium. The final target of this plasma filtration adsorption dialysis is to reach a complete purification, removing hydrophilic and hydrophobic molecules with different ranges of weights [133]. This technique was initially conceived, in the middle of 1990, for sepsis treatment, with the purpose of eliminating pro-inflammatory molecules and cytokines not adequately removed by conventional extracorporeal mechanisms [134]. This method evolved to remove other mediators of inflammation and toxins in several clinical conditions such as liver failure, rhabdomyolysis, cytokine dysregulation, and poisoning [130].

Zhang et al. [135] demonstrated that CPFA rapidly and efficiently alleviated hemolysis in a patient affected by Wilson Disease, because it can also remove copper, bilirubin, and albumin-binding toxins, improving liver injury.

The goal of this treatment is to restore normal immune function by non-selectively adsorbing both pro- and anti-inflammatory mediators [136], and the greatest result is achieved with substances with high-medium molecular weights [137]. CPFA can play a major role for PNH patients by the adsorption of hemolysis products, removal of inflammatory mediators and treatment of AKI, with the concomitant action of CVVH (continuous veno-venous hemofiltration).

## 5. PNH: Current and Future Perspectives of Complement Inhibition Therapy

### 5.1. The Role of the Complement System

The complement system is an archaic maintained component of the innate immune system, consisting of more than 50 serum proteins synthesized as inactive precursors. Under appropriate conditions, they are activated, acting as proteases, cleaving other components consecutively in a cascade to amplify the production of the final effectors [138,139]. Three pathways of complement activation are known: the classic pathway, alternative pathway and lectin pathway. They all result in C3 cleavage to C3a and C3b with the consecutive cleavage of C5 into C5a and C5b. C5a is a strong anaphylatoxin, acting via a distinct C5a receptor (C5aR1, CD88) to recruit leukocytes to areas of complement activation, but it also behaves as a bridge to the adaptive immune system as the receptor is expressed on antigen-presenting and other immune cells [140]. Complement is amplified by the alternative pathway in the case of the absence of complement control proteins on any unprotected surface.

Zewde et al. [141] proposed a model, based on a system of ordinary differential equations, illustrating the mechanisms of the four steps of the alternative pathway under physiological conditions (Table 2).

In PNH, hemolysis is mostly mediated by the alternative pathway of complement, so complement inhibition therapy is the best strategy against this disease. The choices among the different kinds of complement inhibitor or modulator will be soon enriched by several molecules currently under investigation or recently approved. Before the approval of eculizumab, the therapeutic alternative for PNH was only supporting the patients by blood transfusion, iron supplementation, anti-thrombosis prophylaxis or therapy and allogeneic bone marrow transplantation [142].

### 5.2. C5 Inhibitors

The activation of complement factor C5 generates the potent anaphylatoxin C5a, leading to pathogen lysis, inflammation and cell damage [143].

The first C5 inhibitor was h5G1.1.mAb, later named eculizumab. Eculizumab improved the management of and clinical outcomes in patients with PNH, becoming a proof of concept for other complement-mediated diseases.

Eculizumab inhibits the formation of the proinflammatory metabolite C5a and construction of the MAC via C5b, through a mechanism of C5-binding aimed at limiting its cleavage by C5 convertases [144].

The first study that tested eculizumab was the TRIUMPH study [145], which randomized transfusion-dependent PNH patients into groups receiving either the complement inhibitor or placebo. Eculizumab reduced and regulated intravascular hemolysis, with subsequent transfusion-independence in half of the patients, and it was given intravenously at 600 mg weekly for 4 weeks, followed 1 week later by 900 mg every 2 weeks thereafter.

These results were confirmed in the SHEPHERD [146] study, which included a broader population of PNH patients (patients with lower transfusion requirements and with moderate cytopenia were allowed to enter) [147]. Of the 195 individuals entering the pilot, TRIUMPH or SHEPHERD studies, 187 participants completed these steps and were elected to receive eculizumab in a common Phase 3b open-label extension study of 104 weeks. PNH patients treated with eculizumab did not demonstrate a major susceptibility to infections compared to placebo. Prolonged eculizumab therapy was linked to a decrement in hemolysis, lasting over more than 54 months of treatment [148].

Kelly et al. [149] performed a study on 79 patients receiving eculizumab; survival for treated patients was not different from that of age- and sex-matched normal controls, but it was significantly better than in 30 similar patients treated before eculizumab became available [150].

Loschi et al. [151] analyzed the impact of eculizumab treatment on classic PNH in comparison with a non-treated population. Overall, survival at 6 years was 92% (95% CI: 87, 98) in the eculizumab cohort, without increasing the risk of clonal evolution, versus 80% (95% CI: 70, 91) in historical controls diagnosed after 1985 (hazard ratio: 0.38; 95% CI: 0.15, 0.94; *p* = 0.037). For patients diagnosed in the period 1954–1985, overall survival was estimated to be 58% (95% CI: 48, 70).

A recent eculizumab-like monoclonal antibody engineered to have a longer half-life is proposed to guarantee the same effects of eculizumab but with a more advantageous and effective dosing schedule: Ravulizumab (ALXN1210) [6]. Ravulizumab is a long-acting C5 complement inhibitor approved in December 2018 by the U.S. FDA [152] and in July 2019 by the European Commission [153]. This drug represents a new promising instrument for the treatment of PNH, permitting longer dosing intervals of 8 weeks [154].

Ravulizumab is obtained through targeted engineering to incorporate four amino acid substitutions and select adaptations to eculizumab to both control target-mediated drug disposition and boost recycling efficiency through the neonatal Fc receptor [155].

In the largest Phase 3 study in complement-inhibitor-naive PNH patients conducted till now [156], Ravulizumab was demonstrated to be noninferior to eculizumab for all end points: transfusion avoidance (73.6% vs. 66.1%; difference of 6.8% (95% CI: −4.66, 18.14)), LDH normalization (53.6% vs. 49.4%; odds ratio, 1.19 (0.80, 1.77)), percent reduction in LDH (−76.8% vs. −76.0%; difference (95% CI), −0.83% (−5.21, 3.56)), change in FACIT-Fatigue score (7.07 vs. 6.40; difference (95% CI), 0.67 (−1.21, 2.55)), breakthrough hemolysis (4.0% vs. 10.7%; difference (95% CI), −6.7% (−14.21, 0.18)), and stabilized hemoglobin (68.0% vs. 64.5%; difference (95% CI), 2.9 (−8.80, 14.64)).

The study of Kulasekararaj et al. [157] assessed that subjects with PNH may be switched from eculizumab (900 mg administered every 2 weeks) to Ravulizumab (administered every 8 weeks) while maintaining the high level of efficacy, safety and quality of life previously accomplished with eculizumab: percentage change in LDH (difference, 9.21% (95% CI: −0.42, 18.84), *p* = 0.058 for superiority), breakthrough hemolysis (difference, 5.1 (95% CI: −8.89, 18.99)), change in FACIT-Fatigue score (difference, 1.47 (95% CI: −0.21, 3.15)), transfusion avoidance (difference, 5.5 (95% CI: −4.27, 15.68)), and stabilized hemoglobin (difference, 1.4 (95% CI: −10.41, 13.31)).

A subcutaneous formulation of Ravulizumab is under approval [158] and a Phase 3 trial of once-weekly subcutaneous administration of this drug is being planned to support registration for patients with PNH and atypical Hemolytic Uremic Syndrome [159].

There are many other anti-C5 monoclonal antibodies in different stages of development.

RO7112689/SKY59 (Chugai Pharmaceutical/Roche, Tokyo, Japan) is a novel pH-dependent C5-binding antibody for PNH and complement-mediated disorders. It is based on recycling technology: SKY59 gives to the therapeutic antibody a pH-dependent C5-binding function so that C5 is released from the antibody in the acidic endosome and then brought to the lysosome for degradation; at the same time, C5-free antibody returns back to plasma. Sampei et al. [160] recently demonstrated that SKY59 can guarantee a long-lasting neutralization of C5 in animal models, showing potential use for subcutaneous administration or a less frequent therapy regimen. It is also able to provide therapeutic benefit for eculizumab-resistant PNH patients carrying the R885H polymorphism, representing a promising alternative for complement-mediated disorders [161]. Hoffmann-La Roche is currently recruiting, for a Phase 1/2 trial (NCT03157635), healthy volunteers and PNH patients (both treatment-naïve and on eculizumab), who will receive SKY59. Patients with the R885H polymorphism are also investigated by NOVARTIS with another anti-C5 antibody, Tesidolumab/LFG316. This drug is under investigation in a proof-of-concept Phase 2 study enrolling untreated PNH patients [162].

Pozelimab/REGN3918 (Regeneron, Eastview, TN, USA) has been tested in healthy volunteers, showing dose-dependent complement inhibition using a CH50 assay test [163]. Preclinical data and tests on healthy volunteers demonstrated rapid C5 inhibition and a good safety profile [5,164].

Recently, Amgen started enrolling subjects for a randomized, double-blind, active-controlled Phase 3 study evaluating the efficacy and safety of ABP-959 biosimilar, compared with eculizumab, in adult subjects with PNH (NCT03818607) [165]. The primary outcome is hemolysis as measured by LDH in parallel and cross-over comparisons.

Samsung Bioepis is also recruiting patients for a Phase 3 cross-over trial of their eculizumab biosimilar, SB12 (NCT04058158) [166]. Patients will be randomized to receive 600 mg of either the biosimilar or the reference eculizumab through intravenous administration every week for 4 weeks. Then, they will receive 900 mg of eculizumab for the fifth week, and 900 mg every 2 weeks until Week 52. At Week 26, patients will switch between the two groups. The primary outcomes are hemolysis as measured by LDH at Week 26 in a parallel comparison and at Week 52 by a cross-over comparison. The study estimated completion date is July 2021.

Coversin is a recombinant complement inhibitor originating from Ornithodoros moubata that has been demonstrated to be efficient against PNH in an in vitro model [167].

The COBALT study, a Phase 2 single arm open-label trial, demonstrated that Coversin daily subcutaneous injection has a positive safety profile and clinical response in PNH patients with or without the C5 eculizumab-resistant polymorphism, inducing a strong decrease in LDH serum levels [168].

These results demonstrate that Coversin may be an effective alternative even for patients with PNH that prefer the independence of self-administration.

Ra Pharma is developing RA101495/Zilucoplan [169], a synthetic, macrocyclic peptide that binds C5 with a sub-nanomolar affinity and allosterically inhibits its cleavage into C5a and C5b and the assembly of the C5b-9 complex though destabilizing C5b’s interaction with C6. Hill et al. [170] performed a Phase 2 study on Zilucoplan administration in PNH patients enrolled from separate cohorts: one group was on prior eculizumab treatment, and another group included treatment naïve subjects. The latter (*n* = 10) had a pre-specified primary endpoint reached for LDH decrease from baseline to the mean of weeks 6–12 (*p* = 0.002). The effect of this therapy on LDH gave a consistent suppression of complement activity in an ex vivo antibody-sensitized sheep red blood cell direct hemolysis assay. LDH reduction in treatment-naïve patients was also associated with a decrease in transfusion-dependent patients (50% of transfusion-dependent naïve patients achieved transfusion independence after starting therapy) and better quality of life (increase of 5.9 points in FACIT fatigue score) from Week 0 to Week 12.

Zilucoplan also rapidly reduced LDH to the levels seen in patients receiving eculizumab and a complete, sustained and uninterrupted inhibition of complement activity was maintained in the sRBC hemolysis assay before, during and after eculizumab washout [171]. These Phase 2 findings proved that RA101495 may lead to a convenient and cost-effective treatment for PNH patients.

Cemdisiran/ALN-CC5 is another potential tool in C5 inhibition therapy. This is a subcutaneously administered N-acetylgalactosamine (GalNAc)-conjugated small interfering RNA (siRNA) silencing C5 expression in the liver [172]. A Phase 2 open-label study on healthy volunteers and PNH patients [173] showed that the volunteer population had a strong reduction of C5 plasma levels and activity whereas the PNH patients had a slow and partial inhibition of plasma hemolysis and LDH reduction. The addition of eculizumab therapy to Cemdisiran produced a better outcome in patients that are inadequate responders to eculizumab, highlighting a potential role of combination therapy with these two drugs in C5 inhibition treatment [174].

### 5.3. C3 Inhibitors

The clinical challenge associated with managing this complex patient population continues to be addressed. Upstream complement C3-inhibition is an interesting therapeutic option.

This class of complement inhibitors includes the anti-C3 small peptide compstatin with its derivatives, and inhibiting agents of complement factor D and complement factor B.

These molecules offer the possibility of wide-ranging therapeutic strategies for the treatment of the pathogenic processes of complement-related diseases and may therefore play a new pivotal role for further improving the lives of PNH patients [175]. 

Sahu et al. [176], in 1996, discovered compstatin, a cyclic 13-aminoacid peptide C3 inhibitor that over the years paved the way to a family of next generation compstatin analogs.

Compstatin analogs have proven to be promising alternatives for the treatment of complement-related disorders such as sepsis, periodontitis, transplantation, age-related macular degeneration, biomaterial-induced inflammation and PNH [177,178,179].

These drugs bind to a shallow groove assembled between the MG4 and MG5 domains of the β chain of C3, not allowing the interaction of the native protein with C3 convertases [180].

Peptide inhibitors of C3 activation efficaciously regulate hemolysis, uncontrolled C3 activation and the erythrophagocytosis of PNH erythrocytes [181], a process secondary to CD55 absence [179]. The key position of C3 in the complement cascade permits compstatin analogs to prevent both intravascular and extravascular hemolysis [182].

C3, once activated, is cleaved into C3a and C3b: C3b leads to the opsonization of these erythrocytes, causing extravascular hemolysis by the action of white blood cells. This action of C3b, especially in the hepatosplenic compartment, partially explains the persistence of an anemic status and the need for transfusions in PNH patients, despite treatment with a C5 inhibitor [183].

The second generation compstatin analog APL-2 (4(1MeW)7W/POT-4) is a synthetic cyclic peptide conjugated to a polyethylene glycol polymer that specifically binds to C3 and C3b, preventing the activation of all three pathways of complement activation.

APL-2 is currently being evaluated in the PADDOCK [184] Phase 1b trial to examine the safety, preliminary efficacy and pharmacokinetics of subcutaneous administrations of the drug. This molecule is also being studied in the PHAROAH [185] Clinical Trial, a Phase 1 study designed to assess whether APL-2 can provide benefits to patients on treatment with eculizumab that are severely anemic and transfusion-dependent.

A third generation of compstatin analogs is represented by Cp40, which seems to be a fascinating approach to C3 inhibition [186]. Risitano et al. [179] demonstrated in vitro that Cp40 accurately prevents C3 activation and opsonization on PNH erythrocytes; this indicates a possible inhibition of intravascular hemolysis caused by MAC and C3-mediated extravascular hemolysis in vivo. They also tested the long-acting form (polyethylene glycol-Cp40) on the hemolysis and opsonization of PNH erythrocytes, showing that it may be a valid alternative due to its prolonged plasma permanence and strong efficacy.

### 5.4. Factor D Inhibitors

The highly specific S1 serine protease factor D (FD), also known as adipsin, has a fundamental role in the amplification of the complement alternative pathway by catalyzing the cleavage of complement factor B into Ba and Bb, which leads to the production of the AP C3 convertase. FD is considered as an interesting drug target due to its low plasma concentration, high specificity, and pivotal function in AP C3 convertase assembly [175,187].

LNP023 is a novel oral small molecular weight compound that inhibits the alternative pathway and prevents dysregulation on PNH cells in vitro [188]. A Phase 2 study is testing this molecule with the aim of determining its efficacy, safety, pharmacokinetics and pharmacodynamics when administered in addition to the standard of care in PNH patients with signs of active hemolysis [189].

ACH-4471/Danicopan is an oral FD inhibitor that regulates the excessive activation of the complement alternative pathway by inhibiting C3 cleavage, C3 fragment deposition, terminal pathway activation and MAC formation. Risitano et al. [190] demonstrated a strong inhibition of AP action with a maintained modulation of intravascular hemolysis, without the development of C3-mediated extravascular hemolysis.

A Phase 2, open-label trial of Danicopan plus eculizumab in PNH patients reported relevant increases in mean Hb, a reduction in blood transfusions, a meaningful improvement in FACIT fatigue scores versus baseline, a reduction in total bilirubin and mean reticulocytes, and a reversion of LDH into the normal range [191]. A Phase 2 study on PNH patients with inadequate responses to eculizumab is currently ongoing. In this cohort, Danicopan is added to therapy with the purpose of determining the effectiveness of this molecule in improving anemia when given with eculizumab [192].

## 6. Conclusions

PNH is characterized by a plethora of insidious symptoms and damage mechanisms such as hemolysis, peripheral cytopenia, bone marrow dysfunction, thrombosis, arterial and pulmonary hypertension.

Kidney involvement is a common feature of PNH patients, mainly caused by hemolysis and subsequent hemoglobinuria with the progressive deposition of hemosiderin in the renal proximal convoluted tubule. In particular, hemosiderin’s high molecular weight does not allow its complete renal excretion, so it accumulates in tubular epithelium cells, leading to tubular atrophy and interstitial fibrosis. PNH has been rarely associated with glomerulopathies, but the pathogenic mechanisms are still controversial.

Despite the increased knowledge of this syndrome, the most appropriate strategy and choice of therapies for PNH are still up for debate. In the case of chronic hemolysis and renal iron deposition, a multidisciplinary approach should be considered to avoid or treat kidney comorbidities. In this setting, the nephrologist has the task of choosing the most suitable treatment to modulate complement activation and plays a leading role in managing the dangerous case of AKI. Valid options are represented by treatments such as immunoadsorption, dedicated hemodialysis filters that use convective techniques, and backfiltration, or even CPFA.

Further studies are needed, including randomized controlled trials, but the lack of data and univocal indications in some circumstances are so extreme that even single case reports would be informative.

The landscape of combination PNH therapy has markedly progressed over the last few decades, and it will continue to expand with the introduction of long-acting complement inhibitors and the development of complement-modulating drugs.

The choice of the right drug is a key component in the improvement of personalized medical care for patients with such a complex disease. This represents a new set of challenges to providers, hematologists and nephrologists, who are required to keep themselves updated regarding this evolving field.

## Figures and Tables

**Table 1:** Acute tubular necrosis (ATN): different phases.

Initiation Phase	Extension Phase	Maintenance Phase	Recovery Phase
Renal tubular epithelial cell injury	Prolonged hypoxia consecutive to the initial ischemic event	Cells go through repair mechanisms, migration, apoptosis and proliferation with the intent to restore cellular and tubule integrity	Cellular differentiation continues
Renal ischemia	Continued inflammatory response	Epithelial cells provide intracellular and intercellular homeostasis	
Changes in structural and functional alterations in renal PTECs	Cells continue to go through damage and death with both necrosis and apoptosis, principally in the outer medulla	Slowly improving cellular and organ function	Epithelial polarity is reestablished
Alteration of the regular framework of filamentous actin in the cell	The proximal tubule cells in the outer cortex undergo cellular repair and improve morphologically	Blood flow returns toward normal	
Ischemic injury to vascular smooth muscles cells and endothelial cells	GFR continues to fall	GFR is stable at a degree influenced by the severity of the initial event	Normal cellular and organ function is restored. Renal function can be directly linked with the mechanisms of cell injury and recovery
Up-regulation of chemokines and cytokines triggering the inflammatory cascade	Continuous generation and release of chemokines and cytokines		

GFR: glomerular filtration rate; PTEC: proximal tubular epithelial cell.

**Table 2:** Phases of the alternative complement pathway, adapted by Zewde et al. [141].

Phase 1	Phase 2	Phase 3	Phase 4
Initiation (Fluid Phase)	Amplification	Termination	Regulation
Tick-over consists of the hydrolysis of C3 into C3a and C3b in fluid phase, with the activation of the alternative pathway.	C3b molecules can indiscriminately bind to host damaged cells or pathogen surfaces and form C3 convertase that amplifies C3 deposition and initiates a set of cascade reactions.	C3b molecules on the surface of a pathogen lead to opsonization, a process stimulating phagocytosis by macrophages. C3b also binds C3 convertase to form C5 convertase (C3b_2_Bb) of the alternative pathway. This protein complex cleaves C5, resulting in C5a and C5b. C5b initiates the assembly of membrane attack complex (MAC), a pore responsible for cell lysis.	Complement amplification is regulated through the inhibition of convertase formation, dissociation of existing convertases, cleavage of C3b into iC3b and subsequent cleavage of iC3b to C3dg. The terminal pathway is regulated by soluble vitronectin and clusterin, and CD59 complement regulators.

CD59, a membrane inhibitor of reactive lysis.

## Abbreviations

AKI	acute kidney injury
AKIN	acute kidney injury network
ATN	acute tubular necrosis
CF	concentration in the filtrate
CP	concentration in plasma water
CPFA	coupled plasma filtration adsorption
CRRT	Continuous Renal Replacement Therapy
CVVH	continuous veno-venous hemofiltration
CVVHDF	continuous veno-venous hemodiafiltration
DFO	deferoxamine
FD	protease factor D
FDA	Food and Drug Administration
GalNAc	N-acetylgalactosamine
GPI	glycosyl phosphatidylinositol
HD	hemodialysis
KDIGO	Kidney Disease Improving Global Outcome
MAC	membrane attack complex
LDH	lactate dehydrogenase
LPS	lipopolysaccharide
Chronic Kidney Disease	chronic kidney disease
HB	hemoglobin
NF-κB	Nuclear Factor kappa B
MN	Membranous Nephropathy
MW	molecular weights
NO	nitric oxide
Nrf2	nuclear factor erythroid-2-related factor 2
HO-1	Heme oxygenase 1
PIG-A	phosphatidylinositol glycan class A
PNH	Paroxysmal nocturnal hemoglobinuria
PTECs	proximal tubular epithelial cells
RIFLE	risk, injury, failure, loss, end-stage kidney disease
ROS	reactive oxygen species
NaHCO3	sodium bicarbonate
RRT	renal replacement therapy
THP	Tamm Horsfall protein
